# Parenting Stress, Mental Health, Dyadic Adjustment: A Structural Equation Model

**DOI:** 10.3389/fpsyg.2017.00839

**Published:** 2017-05-23

**Authors:** Luca Rollè, Laura E. Prino, Cristina Sechi, Laura Vismara, Erica Neri, Concetta Polizzi, Annamaria Trovato, Barbara Volpi, Sara Molgora, Valentina Fenaroli, Elena Ierardi, Valentino Ferro, Loredana Lucarelli, Francesca Agostini, Renata Tambelli, Emanuela Saita, Cristina Riva Crugnola, Piera Brustia

**Affiliations:** ^1^Department of Psychology, University of TorinoTorino, Italy; ^2^Department of Pedagogy, Psychology, Philosophy, University of CagliariCagliari, Italy; ^3^Department of Psychology, University of BolognaBologna, Italy; ^4^Department of Psychological, Educational and Training Sciences, University of PalermoPalermo, Italy; ^5^Department of Dynamic and Clinical Psychology, Sapienza University of RomeRome, Italy; ^6^Department of Psychology, Catholic University of the Sacred HeartMilano, Italy; ^7^Department of Psychology, University of Milano-BicoccaMilano, Italy

**Keywords:** equation model, parenting stress, dyadic adjustment, parenthood, mental health, perinatal anxiety, post-natal depression

## Abstract

**Objective:** In the 1st year of the post-partum period, parenting stress, mental health, and dyadic adjustment are important for the wellbeing of both parents and the child. However, there are few studies that analyze the relationship among these three dimensions. The aim of this study is to investigate the relationships between parenting stress, mental health (depressive and anxiety symptoms), and dyadic adjustment among first-time parents.

**Method:** We studied 268 parents (134 couples) of healthy babies. At 12 months post-partum, both parents filled out, in a counterbalanced order, the Parenting Stress Index-Short Form, the Edinburgh Post-natal Depression Scale, the State-Trait Anxiety Inventory, and the Dyadic Adjustment Scale. Structural equation modeling was used to analyze the potential mediating effects of mental health on the relationship between parenting stress and dyadic adjustment.

**Results:** Results showed the full mediation effect of mental health between parenting stress and dyadic adjustment. A multi-group analysis further found that the paths did not differ across mothers and fathers.

**Discussion:** The results suggest that mental health is an important dimension that mediates the relationship between parenting stress and dyadic adjustment in the transition to parenthood.

## Introduction

Transition to parenthood is an important time in the lives of individuals, affecting their psychological wellbeing in many ways. Examples include high level of stress because of new parental role (Cornish et al., 2006; Leigh and Milgrom, 2008; Petch and Halford, 2008; Misri et al., 2010; Bornstein and Venuti, 2013; Trillingsgaard et al., 2014), problematic relationship adjustment (Belsky, 1985; Belsky and Isabella, 1985; Belsky et al., 1985; Belsky and Rovine, 1990; Cobb et al., 2008; Lawrence et al., 2008; Garbarini, 2011; Velotti et al., 2011; Zerach and Magal, 2016), and the emergence of anxious and depressive symptoms (Soliday et al., 1999; Matthey et al., 2000; Skari et al., 2002; Buist et al., 2003; Condon et al., 2004; Glazebrook et al., 2004; Goodman, 2004; Edhborg et al., 2005; Schumacher et al., 2008; Figueiredo and Conde, 2011; Fisher et al., 2012; O’Hara and McCabe, 2013; O’Hara and Wisner, 2014; Mazzeschi et al., 2015; Anding et al., 2016; Della Vedova and Matthey, 2016; Prino et al., 2016; Vismara et al., 2016).

Although having a child is a joyful event, it is also characterized by new responsibility and exceptional demands on the new parents (Petch and Halford, 2008; Trillingsgaard et al., 2014). Sometimes the couple is overwhelmed by the changes and feels unable to cope with all the demands that the new role implies (Abidin, 1995; Petch and Halford, 2008; Trillingsgaard et al., 2014). According to Abidin (1995), parenting stress is the discrepancy between the resources required for the parental role and the perception of being able to cope with them. Parents’ and children’s characteristics and their relationship can also lead to parenting stress (Misri et al., 2010). Current literature identifies an association between mental health—defined as depressive and anxious symptoms (Kendig et al., 2017)—and parenting stress, confirming that parenting stress results in depression (Soliday et al., 1999; Leigh and Milgrom, 2008; Gray et al., 2012; Prino et al., 2016; Riva Crugnola et al., 2016; Vismara et al., 2016) and anxiety (Leigh and Milgrom, 2008; Prino et al., 2016; Riva Crugnola et al., 2016; Vismara et al., 2016).

Both mothers and fathers (O’Hara, 2009) can be affected by post-partum depression (PPD), which is the most common mood disorder during the perinatal period (American Psychiatric Association [APA], 2000, 2013). The incidence of PPD in mothers is reported to be in the range of 15–20% (Fisher et al., 2012; O’Hara and McCabe, 2013). According to DSM-5 (American Psychiatric Association [APA], 2013) PPD is typically experienced from 4 weeks to 6 months after delivery; however, in clinical practice and research, this period is known to stretch up to 12 months after the child’s birth (O’Hara and McCabe, 2013). The percentage of incidence in fathers is from 1.2 to 25.5% (Goodman, 2004) but, unlike in mothers, PPD in fathers is delayed and it often follows the disorder in mothers (Matthey et al., 2000; Prino et al., 2016). Literature shows that parenting stress can influence the onset of PPD and vice versa (Soliday et al., 1999; Leigh and Milgrom, 2008; Gray et al., 2012; Prino et al., 2016; Vismara et al., 2016). Leigh and Milgrom (2008) note that PPD represents the most predictive factor of parenting stress. Soliday et al. (1999) consider parental stress the main risk factor in the development of PPD in both parents. Another factor is the presence of anxious symptoms, which are higher during the prenatal period and lower after birth (Buist et al., 2003; Condon et al., 2004; Heron et al., 2004; Andersson et al., 2006; Figueiredo and Conde, 2011). Anxious symptoms prevail on PPD during the entire perinatal period (Wenzel et al., 2005; Lee et al., 2007). Although most of the existing literature on PPD focuses on mothers (Field et al., 2006), the small number of studies on PPD in fathers suggest that mothers have higher levels of anxious symptoms than fathers (Matthey et al., 2000; Skari et al., 2002; Edhborg et al., 2005; Figueiredo and Conde, 2011; Candelori et al., 2015; Vismara et al., 2016). The link between anxious symptoms and parenting stress has been confirmed by reports (Cornish et al., 2006; Leigh and Milgrom, 2008; Misri et al., 2010; Prino et al., 2016). Anxiety at 3 months after child’s birth is related to parenting stress reported at the same time (Prino et al., 2016). Parenting stress can not only influence both parents individually and predict post-natal depression symptomatology in both men and women, but it can also have adverse implications for couples’ functioning (Soliday et al., 1999).

Transition to parenthood may lead to changes in the marital relationship of parents (Spanier, 1979; Hazan and Shaver, 1994; Darwiche et al., 2015), specifically regarding dyadic adjustment (Spanier, 1979), a construct characterized by dyadic cohesion (DAS-DC), troublesome dyadic differences, consensus on important issues related to dyadic functioning and dyadic satisfaction (DAS-DS). Current literature points to the bidirectional correlation between symptoms of depression and dyadic adjustment (Kurdek, 1999; Davila, 2001; Mamun et al., 2009). After the child’s birth, parents may experience a decrease in dyadic adjustment (Mitnick et al., 2009; Darwiche et al., 2015). Studies also show that dyadic adjustment may be strongly associated with parenting stress (Horowitz and Damato, 1999; Ostberg and Hagekull, 2000; Ganiban et al., 2007; Salonen et al., 2010; Stapleton et al., 2012; Mazzeschi et al., 2015). The lack of partner support, lower dyadic adjustment, and the presence of conflict within the couple can also predict post-natal depressive and anxious symptoms (O’Hara et al., 1992; Cox et al., 1999; Whisman et al., 2011; Trillingsgaard et al., 2014; Darwiche et al., 2015).

To date, the relationship between parenting stress, anxious-depressive symptoms, and dyadic adjustment has been examined only in a single study (Gray et al., 2012). This work intends to deepen the knowledge on this relationship by analyzing a larger sample of mothers and their partners. The aim of this study is to investigate the relationships between parenting stress, mental health, and dyadic adjustment among first-time parents. We use structural equation modeling to examine the potential mediating effects of mental health on the relationship between parenting stress and dyadic adjustment. We hypothesize that higher levels of parenting stress are associated with poorer mental health and that both higher levels of parenting stress and poor mental health are associated with less dyadic adjustment. We also predict that mental health mediates the effects of parenting stress on dyadic adjustment. Multi-group analyses were conducted to determine whether the mediation model differed between mothers and fathers.

## Materials and Methods

### Participants

The sample was composed of 268 parents (134 couples) and their 134 healthy 1-year old babies (61% boys and 39% girls). Participation was voluntary, and participants were recruited from neonatology units and family healthcare services in Italy. Of the couples, 80% were married and 20% were cohabiting. In terms of socio-economic status, most parents belonged to the working Italian middle class. The design excluded subjects who had psychiatric or physical diagnoses as emerged through self-report screening and those whose babies presented genetic or organic problems.

### Measures

#### The Parenting Stress Index—Short Form (PSI-SF; Abidin, 1995; Guarino et al., 2008)

Is a self-report instrument that measures stress specifically associated with parenting. The PSI-SF consists of 36 statements that refer to activities completed in the past week. All items are rated on a 5-point scale. The total stress score is a composite score of the subscale scores: parental distress (PSI-PD), parent–child dysfunctional interaction (PSI-PCD-I), and difficult child (PSI-DC). The PSI-PD measures the stress score of the individuals in relation to their parental role. The scale and subscale explore parenting competence, restrictions on life introduced by parenting, parental conflict, depression, and social support. The PSI-PCD-I analyzes the level of stress perceived by parents because of interactions with the child that seem frustrating. The last scale, PSI-DC, measures how a parent rates the child in terms of their relationship: easy or difficult. This scale is related to the child’s temperament. In the current study, the internal consistency coefficient for the mothers was α = 0.93, and for the fathers, it was α = 0.93.

#### The Edinburgh Post-natal Depression Scale (EPDS; Cox et al., 1987)

Is a self-report questionnaire that consists of 10 items addressing depression symptoms occurring within the previous 7 days. The total score is calculated by adding individual items on a 4-point Likert scale. In the current study, the internal consistency coefficient for the mothers was α = 0.80, and for the fathers, it was α = 0.73.

#### The State-Trait Anxiety Inventory (STAI; Spielberger et al., 1983; Pedrabissi and Santinello, 1989)

Is a commonly used self-report measure of trait and state anxiety. STAI has 20 items for assessing trait anxiety (STAI-T) and 20 for state anxiety (STAI-S). All items are rated on a 4-point scale (i.e., from “Almost Never” to “Almost Always”). In the current study, the internal consistency coefficient for STAI-S in the case of mothers was α = 0.94, and for the fathers, it was α = 0.91. The internal consistency coefficient for STAI-T in the case of mothers was α = 0.89, and for the fathers, it was α = 0.86.

#### The Dyadic Adjustment Scale (DAS; Spanier, 1979; Gentili et al., 2002)

Is a 32-item self-report instrument for assessing dyadic or marital adjustment. The total score is a composite score of the subscale scores: dyadic consensus (DAS-DCS), affectional expression (DAS-AE), DAS-DS, and DAS-DC in couples.

The DAS-DCS measures the level of agreement on what is considered important for the relationship, the DAS-AE assesses the level of expression of affection as well as the sexual relationship, the DAS-DS measures the level of satisfaction on the relationship, and the DAS-DC the level of closeness and shared activities between the partners. In the current study, the internal consistency coefficient for the mothers was α = 0.77, and for the fathers, it was α = 0.76.

### Procedure

The research was approved by university ethics committee. All participants signed the written informed consent form. Data were collected approximately at 12 months of the baby’s age. Parents who met the selection criteria and agreed to participate completed the following independently at home: a set of questionnaires about demographics and the PSI-SF, EPDS, STAI, and DAS self-reports.

### Data Analyses

Descriptive statistics (i.e., means, standard deviations, skewness, and kurtosis) were calculated for the psychological variables. One-way ANOVAs examined gender differences on the considered variables. Pearson’s correlations were used to assess the associations between variables. The analysis of the hypothesized mediation model was based on the two-step procedure (Anderson and Gerbing, 1988): in the first step, confirmatory factor analysis (CFA) was used to construct a measurement model with an acceptable fit to the data. In the second step, the established structural model was verified. The hypothesized model comprised one supposed latent antecedent variable (parenting stress), one latent mediator variable (mental health), and one latent outcome variable (dyadic adjustment). The latent variable parenting stress was assessed using the three subscales of PSI (PSI-PD, PSI-PCD-I, and PSI-DC). The mental health latent variable was assessed from three sources: the EPDS, the STAI-S, and the STAI-T of STAI. The dyadic adjustment latent variable was assessed using the four subscales of DAS (DAS-DCS, DAS-AE, DAS-DS, and DAS-DC).

The evaluation of model fit was based on chi-squared plus recommended criteria for a set of fit indices. Comparative Fit Index [CFI] and Tucker Lewis Index [TLI] = 0.90, which indicate a reasonable fit of the model (Bentler, 1990; Schumacker and Lomax, 1996; Kline, 2005; Brown, 2006). The root mean square error of approximation (RMSEA) of 0.05 can be considered as a good fit; values between 0.05 and 0.08 indicated adequate fit (Browne and Cudeck, 1993; Hu and Bentler, 1999; Brown, 2006). The value of the Standardized Root Mean Square Residual (SRMR < 0.1) (Bentler, 1990) was also acceptable. Multi-group analyses were conducted to determine whether the hypothesized model performed equivalently across genders.

## Results

### Preliminary Analysis

Descriptive statistics for the total sample and by gender are presented in **Table 1**. The mean, standard deviation, skewness, and kurtosis of the 10 observed variables were examined to check for normality of distribution. All the skewness and kurtosis values of the 10 observed variables were less than 1.0, except for PCD-I, DC, and STAI-S. In general, the scores from this sample can be characterized as having a normal distribution. However, a square-root transformation was performed for the PCDI-I, DC, and STAI-S variables. Three variables were derived and named PCD-Is, DCs, and STAI-Ss. The skewness and kurtosis for the PCD-Is (1 and 0.57), for the DCs (0.98 and 0.42), and for STAI-Ss (0.68 and 0.53) indicated a normal distribution. The PCD-I and PCD-Is, the DC and DCs as well as the STAI-S and STAI-Ss were highly correlated (*r* = 0.89, *r* = 0.99, and *r* = 0.99, respectively). Thus, PCD-Is, DCs, and STAI-Ss transformed variables were used in subsequent analyses.

**Table 1:** Descriptive statistics for the total sample and by gender.

*Variable*	Total (*N* = 268)	Mothers (*N* = 134)	Fathers (*N* = 134)
**Age**			
*Mean (SD)*	36.6 (5.6)	35.1^∗^ (4.8)	38.2^∗^ (5.8)
*Range*	20–54	20–45	20–54
**Education**			
*Frequency (%)*			
Elementary school education	25 (9%)	5 (4%)^∗∗^	20 (15%)^∗∗^
High school diploma	117 (44%)	54 (40%)^∗∗^	63 (47%)^∗∗^
University degree	91 (34%)	56 (42%)^∗∗^	35 (26%)^∗∗^
Ph.D.	35 (13%)	19 (14%)^∗∗^	16 (12%)^∗∗^

*^∗^Age difference between mothers and fathers *(t* = 4.78, *p* = 0.000); ^∗∗^education difference between mothers and fathers (χ^2^ = 14.80, *p* = 0.002).*

One-way ANOVAs revealed statistically significant gender differences on PSI-PD scores *F*(1;267) = 7.86, *p* < 0.01, partial η^2^ = 0.03; EPDS scores *F*(1;267) = 23.92, *p* < 0.001, partial η^2^ = 0.08; STAI-Ss scores *F*(1;267) = 7.84, *p* < 0.01, partial η^2^ = 0.03; STAI-T *F*(1;267) = 12.15, *p* < 0.01, partial η^2^ = 0.04, and DAS-DC *F*(1;267) = 11.046, *p* < 0.01, partial η^2^ = 0.04. Mothers showed higher parental distress, higher scores on both depressive and anxiety symptomatology, and lower scores on DAS-DC compared to fathers. Means, standard deviations, skewness, and kurtosis for the 10 observed variables of the total sample and by gender are shown in **Table 2**. The correlation coefficients between age, education, and the 10 observed variables are shown in **Table 3**. No significant correlations were found between age or education and the observed variables.

**Table 2:** Means, Standard Deviations, Skews, and Kurtosis for the 10 Observed Variables.

	Variable	*M*	*SD*	Skewness	Kurtosis	Cut-off scores
**Total sample**	PSI-PD	21.8	7.1	0.6	0.0	
	PSI-PCD-I	17.3	4.9	1.4	1.5	
	PSI-DC	19.2	6.3	1.3	1.5	
	TOT-PSI	58.2	15.9	0.9	0.5	>90
	EPDS	3.9	3.5	1.0	0.3	>9
	STAI-S	31.9	8.2	1.1	1.5	>40
	STAI-T	32.6	7.7	0.7	0.0	>40
	DAS-DCS	54.1	6.5	–0.5	0.0	
	DAS-AE	30.6	4.8	–0.3	–0.6	
	DAS-DS	9.4	1.9	–0.6	0.0	
	DAS-DC	16.5	3.2	–0.3	0.7	
	TOT-DAS	111	11.9	–0.4	0.4	<100
**Mothers**	PSI-PD	23	7.3	0.6	–0.2	
	PSI-PCD-I	17.1	4.9	1.5	1.9	
	PSI-DC	19.6	6.6	1.2	0.8	
	TOT-PSI	60	16.3	0.9	0.4	>90
	EPDS	4.9	3.8	0.6	–0.3	>9
	STAI-S	33.3	8.9	1.1	0.8	>40
	STAI-T	34.2	8.1	0.4	–0.5	>40
	DAS-DCS	54.2	6.4	–0.4	–0.3	
	DAS-AE	30.2	4.7	–0.6	–0.5	
	DAS-DS	9	2	–0.6	–0.3	
	DAS-DC	15.8	3.1	–0.5	0.1	
	TOT-DAS	110	11.8	–0.6	0.8	<100
**Fathers**	PSI-PD	20.6	6.7	0.6	0.2	
	PSI-PCD-I	17.4	4.9	1.2	1.3	
	PSI-DC	18.7	5.9	1.5	2.6	
	TOT-PSI	57	15.4	0.9	0.5	>90
	EPDS	2.9	2.9	1	0.9	>9
	STAI-S	30.5	7.3	1.2	2.6	>40
	STAI-T	31	7	0.9	1	>40
	DAS-DCS	53.9	6.7	–0.5	–0.2	
	DAS-AE	31.1	4.8	–0.2	–0.8	
	DAS-DS	9.5	1.8	–0.6	–0.1	
	DAS-DC	17.1	3.2	–0.2	–0.1	
	TOT-DAS	112	12.1	0.2	0.4	<100

*PSI-PD, parental distress; PSI-PCD-I, parent–child dysfunctional interaction; PSI-DC, difficult child; TOT-PSI, total stress scores; EDPS, total scores of the EPDS scale; STAI-S, state anxiety; STAI-T, trait anxiety; DAS-DCS, dyadic consensus; DAS-AE, affectional expression; DAS-DS, dyadic satisfaction; DAS-DC, dyadic cohesion; TOT-DAS, total score of the DAS scale.*

**Table 3:** Correlations between the demographic variables and the 10 observed variables.

	1	2	3	4	5	6	7	8	9	10	11	12
(1) AGE	–											
(2) EDUCATION	0.18ˆ**	–										
(3) PSI-PD	0.01	0.00	–									
(4) PSI-PCD-I	–0.00	–0.09	0.61ˆ**	–								
(5) PSI-DC	0.03	0.04	0.62ˆ**	0.69ˆ**	–							
(6) EPDS	0.05	0.06	0.46ˆ**	0.28ˆ**	0.35ˆ**	–						
(7) STAI-S	–0.01	0.12	0.39ˆ**	0.32ˆ**	0.33ˆ**	0.65ˆ**	–					
(8) STAI-T	0.01	0.07	0.45ˆ**	0.30ˆ**	0.32ˆ**	0.66ˆ**	0.72ˆ**	–				
(9) DAS-DCS	–0.04	0.03	–0.33ˆ**	–0.15ˆ*	–0.20ˆ**	–0.30ˆ**	–0.29ˆ**	–0.41ˆ**	–			
(10) DAS-AE	0.00	0.03	–0.27ˆ**	–0.21ˆ**	–0.25ˆ**	–0.230ˆ**	–0.26ˆ**	–0.29ˆ**	0.30ˆ**	–		
(11) DAS-DS	0.08	–0.07	–0.25ˆ**	–0.15ˆ*	–0.18ˆ**	–0.262ˆ**	–0.31ˆ**	–0.32ˆ**	0.51ˆ**	0.22ˆ**	–	
(12) DAS-DC	–0.08	–0.08	–0.24ˆ**	–0.16ˆ**	–0.16ˆ*	–0.28ˆ**	–0.26ˆ**	–0.28ˆ**	0.38ˆ**	0.21ˆ**	0.40ˆ**	–

*^∗^*p* < 0.05; ^∗∗^*p* < 0.01.*

### Mediation Model

#### First Step: Measurement Model

**FIGURE 1:**
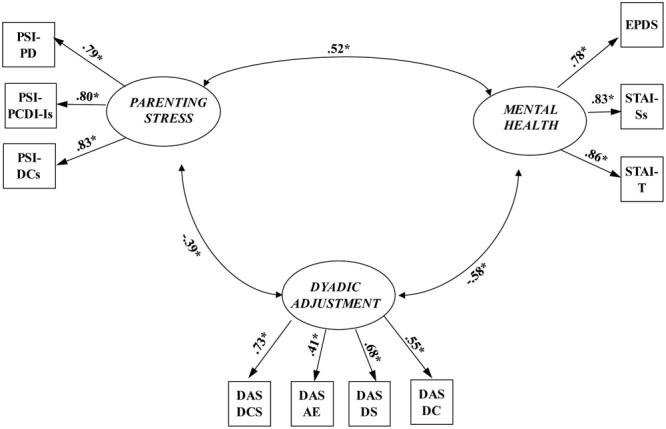
**The measurement model (*N* = 268).** PSI-PD, parental distress; PSI-PCD-Is, parent–child dysfunctional interaction (after transformation); PSI-DCs, difficult child (after transformation); EDPS: total scores of the EPDS scale; STAI-Ss, state anxiety (after transformation); STAI-T, trait anxiety; DAS-DCS, dyadic consensus; DAS-AE, affectional expression; DAS-DS, dyadic satisfaction; DAS-DC, dyadic cohesion; *^∗^p* < 0.001.

The CFA considered the three latent variables and the 10 observed variables (**Figure 1**). All latent variables were agreed to correlate with one another. The measurement model was assessed using the maximum-likelihood method. A test of the measurement model indicated a highly satisfactory fit to the data: χ^2^ = 59.80, *df* = 32, *p* = 0.002, CFI = 0.97, TLI = 0.96, RMSEA = 0.06 (90% [CI]: 0.03 to 0.08), SRMR = 0.05. In addition, all the factor loadings were significant (*p* < 0.001), which confirmed the convergent validity of the indicators (Anderson and Gerbing, 1988). These results indicated that all the latent variables were well represented by their respective indicators (observed variables). In addition, the latent antecedent variable, the latent mediator variable, and the latent outcome variable were significantly correlated with each other (*p* < 0.001). Thus, the measurement model was used to test the hypothetical structural model.

#### Second Step: Structural Equation Model

The structural equation model was tested using the maximum-likelihood method. Testing for mediation effects in structural equation modeling involves the evaluation of three models (Holmbeck, 1997). First in Phase 1, a direct-effect model was used to assess the effect of the predictor (parenting stress) on the outcome variable (dyadic adjustment) in absence of the mediator (mental health). It is necessary to determine that there is a direct connection between the predictor and the outcome variables (parenting stress and dyadic adjustment, respectively). The direct path coefficient from parenting stress to dyadic adjustment was significant (-0.38, *p* < 0.001). Phase 2 involved testing a partial mediation structural model that estimated the direct relationship between parenting stress and dyadic adjustment and added paths from parenting stress to mental health and from mental health to dyadic adjustment. The partial mediation structural model was an appropriate fit: χ^2^ = 59.80, *df* = 32 *p* = 0.002, CFI = 0.97, TLI = 0.96, RMSEA = 0.06 (90% [CI]: 0.03 to 0.08), SRMR = 0.05.

**FIGURE 2:**
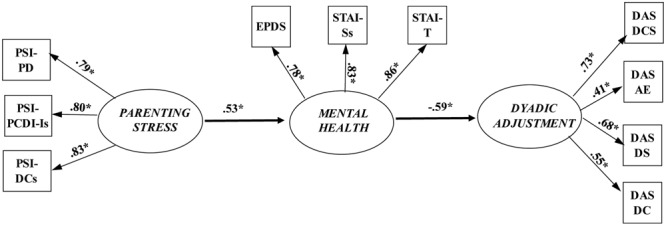
**Fully mediated structural equation model.** PSI-PD, parental distress; PSI-PCD-Is, parent–child dysfunctional interaction (after transformation); PSI-DCs, difficult child (after transformation); EDPS: total scores of the EPDS scale; STAI-Ss, state anxiety (after transformation); STAI-T, trait anxiety; DAS-DCS, dyadic consensus; DAS-AE, affectional expression; DAS-DS, dyadic satisfaction; DAS-DC, dyadic cohesion; *^∗^p* < 0.001.

In Phase 3, the partial mediation model was compared with a full mediation model in which the direct path from parenting stress to dyadic adjustment was constrained to zero. The fit indices for the full mediation model (**Figure 2**) indicated very good fit: χ^2^ = 61.96, *df* = 33, *p* = 0.002, CFI = 0.97, TLI = 0.96, RMSEA = 0.06 (90% CI: 0.04 to 0.08), SRMR = 0.06. Comparison of the chi-squared values indicated no significant difference between the partial and full mediation models, (Δχ^2^ = 2.16, *df* = 1, *p* = 0.14). It should also be observed that there was no significant direct effect of parenting stress on dyadic adjustment in the partial mediation model (*b* = -0.13, *p* = 0.14). Thus, in agreement with guidelines on parsimony (James et al., 2006), the full mediation model was identified as the better fitting model for these data. In summary, the results of this analysis showed that mental health fully mediated the association between parenting stress and dyadic adjustment.

#### Multi-Group Analyses

Multi-group analyses were performed to examine whether the full mediation structural equation model was similar for mothers and fathers. The first phase in these analyses involved assessing the hypothesized structural model with no constraints based on gender; all regression coefficients, correlations, and means were free to take different values for mothers and fathers. This unconstrained model was then compared to models in which various gender constraints were used. The results revealed that an unconstrained model was a slightly better fit to the data [χ^2^ = 83.51, *df* = 66, *p* = 0.07 CFI = 0.98, TLI = 0.98, RMSEA = 0.03 (90% CI = 0.0 to 0.05), SRMR = 0.06] than the constrained model [χ^2^ = 97.41, *df* = 78, *p* = 0.07, CFI = 0.98, TLI = 0.98, RMSEA = 0.03 (90% CI = 0.0 to 0.05), SRMR = 0.08]. Comparison of the models revealed no differences between the unconstrained and the constrained models (Δχ^2^ = 13.90, *df* = 12, *p* = 0.31), implying that the hypothesized model functioned equivalently for both mothers and fathers.

## Discussion

In the last decades, many researchers have analyzed depressive and anxious symptoms during the perinatal period in mother and fathers and their links to parenting stress; however, none seem to have considered these factors in relation to the dyadic adjustment of the couple (Doss et al., 2009; Mitnick et al., 2009; Darwiche et al., 2015). Various studies show that mothers tend to demonstrate sudden declines in relationship satisfaction after birth while fathers show more gradual declines that are not evident until 6 to 15 months after birth (e.g., Belsky and Hsieh, 1998; Grote and Clark, 2001). To understand the processes leading to such perceptions, it is crucial to evaluate such changes against a complex interplay of several variables that may impact the marital relationship, in the course of transition to parenthood.

In line with previous studies, our findings confirm that the level of parental distress and anxious and depressive symptoms appear to be higher in mothers than in fathers (Kim and Swain, 2007; Paulson and Bazemore, 2010; Vismara et al., 2016). Our research shows how mental health—in terms of depressive and anxious symptoms—could be a mediator between parenting stress and dyadic adjustment. The results offered satisfactory confirmation for the hypothesized structural model. Indices of fit indicated that overall the model was a very good fit to the data.

Earlier research has shown linear relationships between parenting stress and dyadic adjustment (Mazzeschi et al., 2015; Prino et al., 2016); however, our results indicate that parenting stress indirectly influences dyadic adjustment through mental health. It has also been shown that the onset of depressive symptoms in both mothers and fathers is influenced by their own levels of anxiety and parenting stress as well as by the presence of depression in their partners (Vismara et al., 2016). In sum, our findings indicate that mental health acts as a mediator of the relationship between parenting stress and dyadic adjustment in both mothers and fathers. In fact, the results offered satisfactory confirmation for the hypothesized structural model. Indices of fit indicated that overall the model was a very good fit to the data.

The results suggest, also, that the relationship between parenting stress and dyadic adjustment is not simply a direct, linear relationship; rather, mental health results to be an important dimension that plays a mediating role.

Our findings highlight the need to consider the complex array of interacting risk as well as protective variables of different nature that may contribute to the development of specific relational and parenting vulnerabilities within each family configuration. Such knowledge can offer targeted indications for more efficacious and family-specific interventions. As is well known, identifying the malfunctioning features in a marital relationship are important because they can impact and be a risk factor for the child’s development (Prior et al., 2000; Gray et al., 2012). In light of this, low dyadic adjustment—characterized by low levels of consensus, DAS-AE, satisfaction, and cohesion—is an indication of malfunction in the couple. To achieve a functional level of dyadic adjustment, our model suggests that is important not only to work on the stress perceived, but also on the anxious and depressive symptoms for both mothers and fathers.

However, the results of the present study should be considered in the context of its limitations. First, we have no data on the couples’ mental health and relationship before birth, which may have had an influence on their parenting stress, mental health, marital satisfaction, or dyadic adjustment after birth. Secondly, the data may not be generalizable: the sample mainly belonged to a medium to high socio-economic status and was non-refereed. We do not know how such variables may interact within different psycho-social contexts. Thirdly, in this study, we used only self-reported tools that are associated with limitations such as inaccurate reporting and social desirability bias. Finally, the participation in the study was voluntary, and the sample may not represent the characteristics of the general population.

Future studies should examine and consider, from a longitudinal perspective, the relation between mental health—in terms of anxiety, depression and other biological or psychological risk factors—and dyadic adjustment and individual perception of parental stress starting with pregnancy. It would be interesting to include an evaluation of protective factors such as the resilience in mothers and fathers. It would also be worthwhile to analyze in depth the relationship between mental health, dyadic adjustment, and parenting stress, focusing on couples receiving group therapy on coping strategies and self-couple perception.

Despite its limitations, the current research increases significantly our understanding of the underlying mechanisms between parenting stress and dyadic adjustment in first-time parents. The study findings present meaningful evidence for the external validity of the mental health-mediated model in Italy. Moreover, the significant path from parenting stress through mental health to dyadic adjustment sheds further light on the complex relationships among these variables. It is likely that mental health improvement programs and training on coping abilities may help the functioning of couples if provided by supporting services to first-time parents.

## Ethics Statement

The research project obtained the approval from University ethics committees in which the research has been conducted (University of Torino, Cagliari, Bologna, Rome, Milano Cattolica, and Milano Bicocca). This study was carried out in accordance with the recommendations of ‘Universities Ethical Committees’ – as written above – with written informed consent from all subjects. All subjects gave written informed consent in accordance with the Declaration of Helsinki.

## Author Contributions

LR prepared the study design, organized the sample recruitment, collected data, and contributed to the writing of the manuscript’s introduction, discussion, and references sections. LP prepared the study design, organized the sample recruitment, collected data, and contributed to the writing of the manuscript’s introduction, discussion, and references sections. CS prepared the data set, performed statistical analysis, prepared the tables, and contributed to the writing of the methods and results sections. LV contributed to prepare the study design, to organize the recruitment of the sample, and to write all sections of the manuscript. EN, CP, AT, BV, SM, VFen, VFer, and EI contributed to the recruitment of the sample and to data collection. LL, FA, RT, ES, and CR contributed to prepare the study design and supervised data collection and the research team. PB contributed to prepare the study design and supervised the research team and contributed to the writing of the manuscript’s introduction and discussion. All authors reviewed and approved manuscript for publication.

## Conflict of Interest Statement

The authors declare that the research was conducted in the absence of any commercial or financial relationships that could be construed as a potential conflict of interest. The reviewer AP and the handling Editor declared their shared affiliation, and the handling Editor states that the process nevertheless met the standards of a fair and objective review.
